# Linking Psychosocial Stress Events, Psychological Disorders and Childhood Obesity

**DOI:** 10.3390/children8030211

**Published:** 2021-03-10

**Authors:** Marta Rojo, Santos Solano, Tatiana Lacruz, José I. Baile, Miriam Blanco, Montserrat Graell, Ana Rosa Sepúlveda

**Affiliations:** 1 Department of Biological and Health Psychology, School of Psychology, Autonomous University of Madrid, 28049 Madrid, Spain; marta.rojo@uam.es (M.R.); santos.solano@uam.es (S.S.); tatiana.lacruz@uam.es (T.L.); miriam.blancoh@gmail.com (M.B.); 2 Department of Health and Psychology, Faculty of Health Sciences and Education, Open University of Madrid, 28400 Collado-Villalba, Spain; joseignacio.baile@udima.es; 3 Department of Child and Adolescent Psychiatry and Clinical Psychology, Hospital Infantil Universitario Niño Jesús, 28009 Madrid, Spain; montserrat.graell@salud.madrid.org; 4 Centro de Investigación Biomédica en Red de Salud Mental (CIBERSAM), 28029 Madrid, Spain

**Keywords:** psychosocial stress events, childhood obesity, weight status, mental health

## Abstract

There is scientific evidence that supports a strong association between early exposure to stressful life events and the presence of health complications throughout adulthood and, to a lesser extent, in adolescence and childhood. The aim of this study was to examine the accumulation of Psychosocial Stress Events (PSE) and the prevalence of mental disorders in children from 8 to 12 years. The association between these factors and child weight measurements was analysed. A cross-sectional study was conducted among 200 children classified by weight status (obesity, overweight and normal-weight). The assessment was carried out in primary care centres and primary schools. An experienced team carried out a structured medical-psychosocial history and a semi-structured interview aimed at identifying an early diagnosis of psychological disorders. Children filled out a questionnaire to evaluate PSE. The obesity group presented the greatest accumulation of PSE and highest prevalence of psychiatric diagnosis, compared to overweight and normal-weight children. To exceed four or more stressful events was positively associated with psychological problems and child body mass index (BMI z-score). A predictive model confirmed the interaction between a larger number of PSE and the occurrence of a psychiatric diagnosis as variables that predispose children by 26.2 times more to increased weight status. In conclusion, the accumulation of PSE in the family, school and social environments of the children was related to greater psychological distress. If not managed, the likelihood of suffering from other health complications, such as excess weight, may increase. It is important to monitor these variables to ensure positive health outcomes while specifically addressing childhood obesity. This is especially relevant for children from a disadvantaged social background and disharmonious family environments.

## 1. Introduction

Stressful life events are quantifiable incidents that exceed biological and psychological personal resources and affect the individual’s well-being [1]. The accumulation of stressful life events is considered a growing field of study. However, there is a continuing controversy over how to define and classify these events [2]. Stressful events can be differentiated according to their nature (e.g., extraordinary, traumatic or normative character), frequency (chronic stressors, one-off events, daily hassles) or intensity [3]. Specifically, Psychosocial Stress Events (PSEs) are defined as non-traumatic major events with an identifiable beginning and end, which involve acute stress in response to changes in a short period (e.g., parental divorce) [2,4]. There is existing research on the association between a greater accumulation of early stressors and more physical and mental health complications in adults [3,4,5]. Likewise, there is also growing evidence for this association in adolescents and, to a lesser extent, in children. Indeed, previous studies have found that higher rates of stressful life events reported by children were associated with higher scores in psychological symptoms in children and adolescents, such as externalizing and internalizing behaviours (problems of anxiety, depression or somatization, low self-esteem, behavioural problems or cognitive dysfunction) [3,4,6,7,8,9,10]. Similarly, available data have supported this association with the likelihood of presenting a mental disorder, such as Attention Deficit Hyperactivity Disorder (ADHD) and the onset of an Eating Disorder (ED) [10,11], which in turn has been linked to obesity.

However, little is known about the accumulation of PSEs and its relationship with childhood obesity to draw generalizable inferences [12,13]. Childhood obesity is a global health emergency that has presented alarming prevalence rates over the last decades. It has become an increasingly dominant reason for consultation in Primary Health Care Systems. Scientific evidence has reported a tendency in obesity to remain throughout the person’s life. It has also been associated with important short and long-term physical and mental health complications [14,15,16]. Recent theoretical models have highlighted obesity as a complex and multifactorial health problem. In this sense, understanding the risk factors for the development of excess weight in childhood requires a holistic approach involving several personal, family, emotional and socio-cultural variables [17,18].

As part of this approach, environmental stressors have been considered as risk factors related to weight gain and consequently, obesity [5,12,13,14,19]. Indeed, a recent review has pointed out an association between higher adverse childhood experiences (ACE) scores, which are defined as traumatic events (e.g., sexual or physical abuse, domestic violence), and higher rates of excess weight in children from 6 to 12 years [13]. More specifically, previous research has shown that presenting ≥ 4 ACE had a significant impact on the medical and psychological health status in adults, as well as in early and middle childhood. In paediatric samples, exposure to four or more ACE categories was associated with clinical obesity (Body Mass Index BMI ≥ 95th percentile) [20,21,22,23,24].

On the other hand, evidence has supported that the disruption caused by these stressful events increased the risk of poor childhood mental health and emotional distress [5,6,25]. According to Hemmingsson’s proposal, greater social disadvantages (e.g., low-income households or social adversities) without adequate resilience and personal resources may promote psychological vulnerability and an emotional overload in children and their families. In line with this approach, several studies have indicated a higher prevalence of psychological problems in children with overweight and obesity [26,27,28]. This scenario may trigger weight gain-inducing effects, increasing the risk of weight gain and subsequent obesity through the mediating role of child self-regulatory, maladaptive coping strategies or disrupted homeostasis of the child’s organism (e.g., levels of cortisol, leptin and fat percentage) [18,29,30,31].

On the other hand, overweight people also tend to report high rates of weight bias, social stigma, teasing and rejection from peers. These negative experiences have been shown to correlate with low self-esteem, poor emotional-well-being, high BMI and disordered eating behaviours, thus creating a vicious circle [32,33,34,35]. At the same time, all these factors also appear to increase the probability of developing an ED. Likewise, there is an ongoing two-way relationship between both problems, making the presence of one of them a risk factor for developing the other [36].

In conclusion, there is increasing interest in PSEs as relevant factors for health concerns. Most studies have been focused on weight gain over the long-term, and the number of studies that have specifically addressed childhood obesity is small compared to all the available research on other health problems during infancy. Therefore, further research on this topic is warranted. With this in mind, the purpose of this study is to examine the association between PSEs, obesity and related mental health indicators in middle childhood (8–12 years).

Hence, the underlying objectives were (1) to describe the accumulation of PSEs in family, school and social environments and the prevalence of psychological problems in 200 children classified by weight status; (2) to examine whether the exposure to four or more PSEs were associated with a higher prevalence of childhood psychiatric disorders and higher weight status, compared to the group of children who accumulated 0–3 PSEs, and (3) to analyse the association of PSEs and psychological problems in childhood obesity as predictors of higher child body mass index (BMI z-scores) in children.

The hypotheses were the following: (a) children with obesity would present a higher number of PSE, compared to overweight and normal-weight groups; (b) presenting four or more PSEs would be associated with higher probabilities of presenting a mental disorder and higher weight status (BMI z-score); (c) a greater accumulation of PSEs could predict higher probabilities to present a psychological disorder, and both variables in interaction would increase the vulnerability of excess weight during childhood.

## 2. Materials and Methods

### 2.1. Participants and Procedure

This study is comprehended within a larger cross-sectional study designed to identify bio-psycho-family markers for childhood obesity (PSI2011-23127). The sample consisted of 200 Spanish children aged 8 to 12 (M = 10.34; SD =1.31), classified into three groups by their status of weight: obesity group (OBG; *n* = 85; ≥97th percentile), overweight group (OWG; *n* = 65; percentile > 85–97th) and a normal-weight group (NWG; *n* = 50; percentile 15–85th), according to age and sex-specific cut-off points [37,38]. Exclusion criteria were ages out of range (younger than 8 and older than 12), underweight children (children’s weight lower than 15th percentile), secondary obesity (e.g., due to Cushing syndrome), pharmacological treatment that may affect weight and children who did not have an adequate oral or written command of Spanish or suffered from a developmental disorder (e.g., autism spectrum disorders or intellectual disability). Power estimates were calculated using G*Power, version 3.1.3.

Participation was voluntary and did not receive remuneration. The OBG and OWG samples were recruited from several primary care centres in Madrid through flyers provided at routine check-ups by their paediatricians and nurses, who were responsible for establishing the first contact with the families and carrying out the anthropometric measurements. None of the children with excess weight were seeking treatment for obesity at the time of the study. The normal-weight children were recruited from several primary public and charter schools in Madrid through direct response flyers. The recruitment was conducted over a period of four years (2012–2016).

Each family signed an informed assent and consent form before the assessment. A trained interviewer completed a semi-structured interview with the primary caregiver and the child separately. After the interview, the participants also completed a battery of questionnaires.

All the staff members were trained in the administration of the protocol and the assessment interview procedure. There were weekly team meetings with the study coordinator (A.R.S.) to supervise the medical-psychosocial reports and clinical diagnoses of the children. Subsequently, a psychological report was provided to all families participating. Details of the recruitment and sample selection procedure have been published elsewhere [34]. The ANOBAS study received ethical approval by the Niño Jesus Children’s Hospital (Nº. Ref. 0009/10), Primary Care Commission (Ref. 11/12) and the corresponding University Committee (UAM, CEI 27-673/ 76-1394).

### 2.2. Instruments of Assessment

▪ Children’s anthropometric data. Height and weight were measured using a digitally calibrated scale (Type SECA 799 and 769) and a tallimeter at the primary care centres. Child weight status was calculated according to BMI standard deviation scores (BMI z-scores). BMI z-scores were computed by comparing the children’s BMI scores with the ideal BMI of the general population of the same age and sex [36] The average range of z-scores in the whole sample was between −0.33 (SD = 0.76) and +3.08 (SD = 0.83).
▪ Socio-demographic variables. Trained staff carried out a structured medical-psychosocial interview (information about age, marital status, level of education, job occupation and medical and psycho-family history) with the children and their primary caregiver. Families’ socioeconomic status (SES) was calculated using the Hollingshead index [39].
▪ Psychological distress. The prevalence of child psychological disorders in the sample was assessed by trained interviewers through a semi-structured interview to diagnose current and past episodes of psychopathology in children and adolescents according to the DSM-5 (K-SADS-PL, 2018) [40]. The interview was conducted first with the children, and then the primary caregiver confirmed the information. For the current study, we used two categories: 0 (absent disorder) and 2 (definitive clinical disorder).
▪ Psychosocial Stress Events (PSEs). This scale is a new version created ad hoc after a comprehensive systematic review of common stressful events for primary school-aged children (8 to 12 years old). The inventory by Oliva et al. (2008) served as the main reference [7]. A panel of five evaluators was created to check the validity of the structure and content of the items proposed in this new version. The inter-rater reliability of the new questionnaire was calculated by administering the Free Marginal Kappa (*k*) statistical tool among five evaluators. Kappa scores higher than 0.90 were obtained for all the items except one (*k* = 0.60), which was eliminated from the original version. A pilot study was carried out to verify that it was understandable for children between 8 and 12, with adequate responses. Finally, the scale consists of 27 items that comprises three subscales related with adversities to personal (e.g., medical condition), family (e.g., parent’s divorce, serious illness of a relative, sibling rivalry) and school and social environments (e.g., class year repetition, change of school, bullying) during school years. There are two categories of response by the children, depending on whether or not each event occurred (Yes/No). The instrument obtained satisfactory internal consistency of 0.83. The total score corresponds to the sum of the number of events reported by the child. Higher scores indicate a higher number of stress events (PSEs) [41].

### 2.3. Statistical Analysis

Data were analysed using the statistical software package SPSS 24.0 for Windows. Means (M) and Standard Deviations (SD) were used to describe quantitative data and percentages for categorical variables. The three groups by status of weight were compared using multivariate analyses of covariance. Post-hoc Bonferroni comparisons for continuous variables and Chi-square tests (χ^2^) for categorical variables were used. Partial correlations were calculated, controlling for sex and age. Linear and binary logistic regressions were carried out. All *p* values were two-tailed and statistical significance was set at *p* < 0.05. In the questionnaire, a Kappa Coefficient (*k*) was calculated as the proportion of concordance observed over the total number of observations, excluding the concordances attributable to chance. A Kappa index ≥ 0.70 indicates adequate interrater agreement among those observed.

## 3. Results

### 3.1. Anthropometric and Sociodemographic: Characteristics of the Sample

The anthropometric and sociodemographic characteristics of the sample are summarized in Table 1. All 200 participants were aged 8 to 12 (M = 10.34; SD =1.31). Of the total sample, 53.5% of the total sample were females and 46.5% were males. All the primary caregivers who participated were mothers. The family’s socioeconomic status (SES) was calculated according to Hollingshead’s index [39]. There were no significant differences between groups, except for the child’s sex and the mother’s education level (*p* < 0.05).

### 3.2. Differences in Accumulation of Psychosocial Stress Events and Psychological Problems in children by Weight Status

#### 3.2.1. Accumulation of Psychosocial Stress Events by Weight Status

The PSE mean for the whole sample was 2.47 (SD = 2.13) in a range between 0 and 9 events. The highest accumulation of PSEs was obtained in the group of children with obesity (OBG) (M = 3.25; SD = 2.25), followed by the group with overweight (M = 2.36; SD = 1.82) and normal-weight (M = 1.26; SD = 1.69) (F = 15.88, *p* < 0.05). Post hoc analysis showed significant differences in all of the comparisons carried out between the three groups (*p* < 0.05). Sex was entered as a control variable in all the analyses, and no significant differences between males and females in PSE accumulation (*p* > 0.05) were found. On the other hand, the highest percentage of children exposed to four or more PSEs was obtained in the OBG, compared to the OWG and the NWG (χ^2^ = 15.28; *p* < 0.001) (Table 2).

#### 3.2.2. Psychological Distress by Weight Status

Out of the total sample, 47.5% of the children (*n* = 95) met the clinical criteria for a child psychiatric diagnosis according to the DSM-5. More specifically, the percentages of children diagnosed in each group were 69.41% (*n* = 59) in the OBG, 53.84% (*n* = 35) in the OWG and 2.0% (*n* = 1) in the NWG. Significant differences were found between all groups (χ^2^ = 58.92, *p* < 0.001) (Table 2). Anxiety disorder was the psychiatric problem most frequently diagnosed (50.5%; *n* = 48), followed by diagnoses of disruptive, impulse control and conduct disorders (15.8%; *n* = 15); affective disorders (13.7%; *n* = 13); eating disorders and mixed disorders (comorbid anxiety and affective symptomatology) (8.4%; *n* = 8) and elimination disorders (3.2%; *n* = 3).

### 3.3. Association between Psychosocial Stress Events, Weight Status and Childhood Mental Disorders

According to the results, a higher number of PSEs was associated with higher BMI z-scores. More specifically, those children who accumulated a higher number of stressful events were 12.8 times more likely to present higher BMI z-scores (F = 29.44; B = 0.364; *p* = 0.001). In this study, exceeding four or more PSEs explained 7.2% of the child’s weight status (F = 15.99; B = 0.95; *p* < 0.001). Secondly, correlation analyses confirmed a positive correlation between the number of stressful events that the children experienced and the psychological symptoms they reported (*p* < 0.05). Out of 95 children who presented a psychiatric diagnosis in the total sample, 44.2% accumulated four or more PSEs, contrasting with 14.3% in the group of children without psychological problems (*n* = 105). Significant differences were found between both groups (χ^2^ = 21.92; *p* < 0.001). Those children who accumulated ≥ 4 PSEs were more vulnerable to presenting psychological problems in early stages (χ^2^ = 23.96_,_ *p* < 0.001), raising the probability by up to 4.83 times more than participants who presented 0–3 PSEs. In this model, a percentage rate between 13 and 16 of mismatch reduction was obtained with Nagelkerke and Cox–Snell statistics, and 66% of the cases were correctly classified. To analyse the goodness of fit, a Hosmer–Lemeshow test was applied, which concluded that data might fit the model (*p =* 0.99) (Table 3). Sex was entered as a variable but there was no significant effect on the regression model (*p*< 0.05).

### 3.4. Predictive Model of Psychosocial Stress Events on Status of Weight via Psychological Problems: Multiple Linear Regression Model

According to our third aim, a multiple linear regression analysis was tested to estimate children’s weight status (BMI z-scores) based on the following predisposing factors: the accumulation of psychosocial stress events and the presence of psychological disorders in children. The interaction between both variables helped to predict higher weight status (BMI z-scores) explaining 26.2% of the variance (*p* = 0.001). According to this result, the total score of PSEs and the presence of a child psychiatric diagnosis were associated with higher scores of child body mass index (BMI z-scores). In the current study, the presence of a child psychological disorder was a stronger predictor of higher status of weight (R^2^ = 22.9% of the outcome) (F = 58.65; *p* < 0.001) (β-coefficient value = 0.48; t = 7.66; confidence intervals (95% CI): 1.11–1.88; *p* < 0.001), compared to the total number of PSEs (R^2^ = 12.8% of the outcome) (F = 29.44; *p* < 0.001) (B = 0.36; t = 5.43; CI: 0.16–0.36; *p* < 0.001). A sex variable was excluded in all of the above-described models as it was not significant (*p* > 0.05) (Table 4).

## 4. Discussion

The general aim of this study was to examine the accumulation of PSEs in the main environments of a primary school-aged child and the prevalence of psychological disorders as possible predisposing factors of childhood obesity. In our sample, there were three groups of children classified by weight status (obesity, overweight and normal-weight).

Our first hypothesis was confirmed. As expected, children with obesity obtained a larger accumulation of PSEs compared to overweight and normal-weight children. In the current study, PSE average scores were slightly lower than in other studies carried out with adolescents [4,7]. This finding suggests that in our sample the accumulation of stressful events may increase in following years, as adolescence tends to be a stage characterised by many vital changes. This consideration is important, as there appears to be enough evidence to conclude that the accumulation of stressful events may be a major risk factor for later health complications. On the other hand, the group of children with obesity obtained a higher prevalence of psychological problems, finding significant weight-related differences between the three groups (OBG, OWG, NWG). These results are supported by previous research on the association between early exposure to stress and health complications during childhood, finding differences between clinical and general samples [8,32,34]. Previous literature also supports the finding that children with obesity tend to present greater psychological burden than normal-weight children [26,27,28]. In this study, it is necessary to note that 69.41% of the children with obesity presented a psychiatric diagnosis at the time of assessment, compared to 2.0% obtained in the normal-weight group.

Our second hypothesis was partially confirmed. According to our results, there was a positive association between psychosocial stressful events reported by the children and the psychological symptoms that they reported. More specifically, children who accumulated four or more PSEs were more vulnerable to present psychological problems compared to those who presented 0–3 stressful events. These results reiterated the disruption of PSEs on mental health that has been previously reported in the literature [5,6,9]. On the other hand, the accumulation of a higher number of PSEs was found to be a predictor of a higher weight status in the children, thus finding a positive association between both variables (stressful events and higher BMI z-scores). To exceed the risk cut-off point (≥4 PSE) only explained a minor percentage of the child’s weight status, compared to the figures obtained taking into account the number of PSEs as a continuous variable. There are several possible explanations for this result: one may be that increases in overweight measures in response to stressful life events do not appear to occur instantaneously [13]. Previous research has found an association between the exposure to four or more ACEs and clinical obesity (95th percentile) [21,22,23]. Nevertheless, these traumatic events are a different type of event than PSEs, and their longer-term impact could be greater. Even though a predominant psychosocial approach placed the emphasis on the cumulative effect of related stressful events, stress always implies a dynamic relationship between the individual, in this case the child, and the environment. Therefore, further research using both quantitative and qualitative perspectives on PSEs is warranted. From a qualitative approach, it is important to assess the subjective valuation of the different adversities as well as the coping abilities and the resilience of the children and their families [4,7,8].

Accumulation of adverse life events and childhood overweight measures are positively associated. However, increases in overweight measures in response to adverse childhood events do not seem to occur instantaneously. The findings of this study concluded that higher levels of psychological overload were associated with higher number of adversities in the family, school and social environment of the children. In this sense, our third hypothesis was confirmed. The interaction between the accumulation of the stressful events reported by the children and the presence of psychological problems predicted higher weight measures (BMI z-scores). Thus, children who lived in an environment with a greater number of PSE categories, and in addition presented psychological difficulties, were vulnerable to present higher BMI z-scores. This is especially significant when children do not have enough personal skills or adaptive strategies to deal with obstacles and emotional distress. Therefore, we found that children with obesity presented higher prevalence of PSEs and mental disorders, compared to overweight and normal-weight children. In turn, according to previous literature, excess weight during childhood also promoted lower body esteem, worsened psychological well-being, psychiatric comorbidity, social rejection, higher levels of pathological eating attitudes and greater exposure to negative experiences such as teasing [32,33,34,35]. In our study, 48.2% of the OBG participants reported bullying and teasing from their peers, followed by 43.1% and 2.0% of participants in the OWG and the NWG, respectively. Moreover, previous literature has concluded that children with obesity had worse emotional regulation strategies, which in a stressful environment may favour the development of psychological problems and weight gain in the medium term [18,30]. In this study, child psychological distress played a key role, as it explained on its own 22.9% of the child weight status. A psychiatric diagnosis may allow one to identify these psychological difficulties, which in turn may allow for the provision of better coping strategies.

However, the cross-sectional design of the study does not allow us to infer cause-effect relationships. This was one of the main limitations of the study, along with the current controversy about the unclear definition and classification of what are considered stressful life events. Other studies have pointed out the absence of homogeneous and appropriate instruments with which to evaluate them [13]. In particular, the number of questionnaires in Spanish to assess stressful life events is limited, and they are not specific for evaluating the presence of PSEs at school age (6–12 years). All of this has made it difficult to draw firm and generalizable conclusions.

This study has several strengths. Most of the previous research has studied retrospectively the exposure to traumatic events and its impact on adult’s health, and childhood has been scarcely explored. Thus, further investigation in this field is clearly warranted. This study is one of the few that compares PSE accumulation between clinical and general samples in children (8–12 years) classified by weight status. Contrary to the usual way of proceeding in previous research, this study included a self-reported PSE questionnaire answered by the children rather than by their parents. It also incorporated a rigorous protocol to measure weight status and a semi-structured interview with family and children, rather than relying solely on self-reported data. According to previous research, children with obesity presented poor emotional identification, and therefore children’s bias may be present when answering the questionnaires. It is therefore pertinent to carry out a standardized interview that helps to make reliable clinical diagnoses [27]. In particular, the K-SADS diagnostic interview has proven to be an appropriate assessment tool for childhood clinical diagnoses.

For future research, it would be interesting to analyse the possible moderating factors between psychosocial stressors, psychological well-being and weight gain. Unhealthy parenting practices and higher levels of child disordered eating symptoms have been found at disruptive households. These variables were associated with higher child BMI and increased the probability of developing childhood obesity [12,13,24,42,43]. On the other hand, research found high rates of maladaptive eating behaviours (e.g., loss of control eating episodes) as coping strategies associated to unpleasant emotions regulation and psychological distress in children with obesity, promoting weight gain in the medium term [27,28,29,30,31]. Weight gain may either by triggered by the activation of the hypothalamic, pituitary, adrenal (HPA) axis, a disruption in the homeostasis of the child’s organism or by the use of maladaptive strategies [18].

In conclusion, as this study has shown, physical and mental health can be affected before adulthood. Childhood obesity is a global health emergency. However, weight-focused interventions showed only low–moderate effects. An appropriate response to this problem requires a multifactorial and multidisciplinary heath intervention. Improvement efforts to reduce overweight and obesity rates during childhood imply understanding the psychosocial variables related to childhood obesity. This study covers two important factors that matter for childhood obesity: the cumulative effect of PSEs and the presence of child psychological disorders as predictive variables of higher weight status in children from 8–12 years. In this manner, apart from periodic monitoring of anthropometric data from the child population and the promotion of healthy eating and physical activity habits, assessment and intervention strategies should include a regular monitoring of the accumulation of PSEs as well as teaching children new strategies to regulate their unpleasant emotions and coping strategies to deal effectively with stress. Moreover, it is important to identify the most vulnerable groups, for example, through an early detection of risky exposure to adverse conditions within their families, at school and other social environments, rather than waiting to address obesity and its consequences. This new approach may result in better child health. If we are to act effectively and in good time, more in-depth research is needed to support these measures, including longitudinal and follow-up studies.

## Figures and Tables

**Table 1:** Descriptive characteristics of children and their families. Comparisons by groups.

	OBG (*n* = 85)	OWG (*n* = 65)	NWG (*n* = 50)	F/ᵪ^2^(*p*) *
**Children variables**				
**Age** (M (SD))	10.38 (1.34)	10.45 (1.19)	10.14 (1.48)	0.84
**Sex** (% (*n*))				15.76 **
Male	40.0 (34)	66.2 (43)	32.0 (16)	
Female	60.0 (51)	33.8 (22)	68.0 (34)	
† BMI z-score (M (SD))	3.08 (0.83)	1.35 (0.46)	−0.33 (0.76)	371.84 **
**Family variables**				
Civil status (% (*n*))				0.32
Married/Living together	70.7 (58)	73.3 (449)	70.0 (35)	
Divorced/Separated/Single	28.0 (23)	25.0 (15)	28.0 (14)	
Widowed	1.2 (1)	1.7 (1)	2.0 (1)	
**Mother’s level of education** (% (*n*))				9.51 *
No studies/Primary	8.6 (7)	5.0 (3)	-	
Secondary/Baccalaureate	70.4 (57)	56.7 (34)	69.4 (34)	
Higher Ed./University/Postgraduate	21.0 (17)	38.3 (23)	26.5 (13)	
**Mother’s work situation** (% (*n*))				3.93
Full-time/Part-time Work	78.0 (64)	64.4 (38)	67.3 (33)	
Unemploy/Medical leave/Retirement	15.9 (13)	23.7 (14)	24.5 (12)	
Housework	6.1 (5)	11.9 (7)	8.2 (4)	
**SES** (% (*n*))				14.28
I _Lowest_	7.5 (6)	12.9 (8)	4.0 (2)	
II	21.3 (17)	12.9 (8)	12.0 (6)	
III	46.3 (37)	41.9 (26)	50.0 (25)	
IV	17.5 (14)	17.7 (11)	32.0 (16)	
V _Highest_	7.5 (6)	14.5 (9)	2.0 (1)	

Note: † BMI z-score standard deviation scores were computed by comparing the subject’s body mass index with the ideal BMI of the general population of the same sex and age. NWG = normal-weight group; OBG/OWG = obesity and overweight group; SES = socioeconomic status; M = mean; SD = standard deviation. In bold, * p-values < 0.05, ** *p*-values < 0.001.

**Table 2:** Accumulation of Psychosocial Stress Events (PSEs) and differences in children by weight status.

	OBG (*n* = 85)	OWG (*n* = 65)	NWG (*n* = 50)	F/ χ^2^ Overall *p*-Value	Post Hoc *p*-Values
Total PSE M (SD)	3.25 (2.25)	2.36 (1.82)	1.26 (1.69)	15.88 ***p* < 0.001**	***p* < 0.001**
Cut-off point ≥ 4 PSE % (*n*)	41.2 (35)	26.2 (17)	10 (5)	15.28 ***p* < 0.001**	-
Prevalence of psychiatric diagnoses % (*n*)	69.41% (59)	53.84% (35)	2.0% (1)	58.92 ***p* < 0.001**	-

Note: PSE = psychosocial stress events; NWG = normal-weight group; OBG/OWG = obesity and overweight group; M = mean; SD = standard deviation. Bold means significant value at *p* < 0.05.

**Table 3:** Binary logistic regression analysis: risk prediction model of psychological disorders.

Model	B	Exp (B)	*p*
Factor 1: ≥4 PSE	1.58	4.83	**0.001**
Factor 2: Sex	−0.07	0.93	0.229

Note: Dependent variable: psychological problems. In bold, significant values at *p* < 0.05.

**Table 4:** Linear regression model on children weight status (BMI z-scores).

Predictors on BMI z-Score	F (*p*)	R^2^ Adjusted	β	t (*p*)
PSE Total	**3.10 (0.01)**	-	0.21	**35.44 (0.001)**
Interaction PSE & Presence of a psychiatric diagnosis	**6.01 (0.001)**	0.262	0.40	6.01

Note: In bold, F: ANOVA F-statistic; R^2^: coefficient of determination; β: coefficient value; *t*-value; *p*-value < 0.05 values.

## Data Availability

The data presented in this study are available on request from the corresponding author. The data are not publicly available due to privacy and ethical reasons (personal data relating to under age users).
